# Formulation, Characterization and Optimization of β–Glucan and Pomegranate Juice Based Films for Its Potential in Diabetes

**DOI:** 10.3390/nu14102142

**Published:** 2022-05-20

**Authors:** Ionut Avramia, Sonia Amariei

**Affiliations:** Faculty of Food Engineering, Stefan cel Mare University of Suceava, 720229 Suceava, Romania; sonia@usm.ro

**Keywords:** β–glucan, pomegranate juice, bioactive films, diabetes, vasoprotective effects

## Abstract

The aim of this study was to develop films based on β–glucans in association with pomegranate juice for its potential in metabolic disorders such as diabetes due to plenty of bioactive compounds from the film composition. Initially, a Box-Behnken design was generated by varying the level of β–glucan content (0.5, 1, 1.5 g), sodium alginate (0.2, 0.4, 0.6 g) and pomegranate juice (10, 20, 30 mL) for development of films. Subsequently, glycerin was added as 25% of the total dry matter. The optimization of the films prepared by the solvent casting method was conducted based on the different responses such as: water vapor transmission rate (WVTR), water vapor permeability (WVP), thickness, density, moisture content, solubility, film opacity and color. The water activity profile and FT–IR analysis were performed in all tests. The model was used to determine the optimal experimental values considering that the optimal film will make a sustained contribution to diabetes. The optimal values of the film sample made of β–glucans, sodium alginate, pomegranate juice and glycerin make it befitting for packaging dry powdered pharmaceuticals. Finally, antimicrobial activity against Gram-negative and Gram-positive bacteria, UV barrier properties and microcrack and pore detections through SEM were also investigated for the optimal film sample.

## 1. Introduction

Diabetes or “diabetes mellitus” is a part of metabolic diseases characterized by hyperglycemia determined by poor insulin secretion, insulin action or both. Until now, four types of diabetes were known: Type I diabetes or insulin-dependent in which the body no longer produces insulin, type II diabetes caused by the inability of the pancreas to produce enough insulin needed by the body or the resistance of cells to its actions [1], gestational diabetes which is common in pregnant women and disappears after pregnancy [2] and type III diabetes, first introduced in 2005 which is associated with Alzheimer’s disease and has the characteristics of both type I and type II diabetes [3].

The slow progression of hyperglycemia is associated with long-term damage, functional disorders of the organs and deterioration, especially in the eyes, kidneys, nerves, heart and blood vessels [4]. Related complications in diabetes due to an inadequate glycemic control range from retinopathy, renal sclerosis to obstruction of blood vessels in legs, coronary, cerebral system or neuropathy [5,6].

In 2021, Arora et al. (2021), through a comprehensive study of diabetes medication, showed a new perspective in the transition from synthetic drugs to herbal therapies. Together with the collaborators, they highlighted the bioactive compounds in plants and the high potential that medicinal plants can play in the treatment of diseases such as the case of diabetic neuropathy [7]. In this context, finding bioactive substances and formulating bioactive films that would bring a sustained contribution in chronic diseases related to diabetes would be beneficial.

Bioactive films belong to edible films and are made by incorporating functional ingredients or active agents which may provide antimicrobial or antioxidant properties [8,9]. The bioactive substances are blended directly into the film-forming solution or by sprinkling [10]. In numerous studies, films are used for food packaging, and the trend is to develop active and intelligent packaging systems [11]. The main role of the films is intended for product safety, maintaining the quality of food and allowing a usability enhancement [12]. The advantage of films is that they can be used as a drug delivery system considering their significant stability of the solid in combination with a good applicability in the liquids forms [13]. Apart from the great solubility properties, different inactive compounds such as excipients and fillers are eliminated by the use of films instead of supplements.

Films containing β–glucan, pomegranate juice and sodium alginate are one of these types of bioactive film which can help in diabetes complications.

Yeast β–glucan is a type of glucose polysaccharide which has a backbone structure of β–1,3-glucan interconnected by β–1,6 chains [14]. The β–1,3/β–1,6 linkages provide a triple helix structure responsible for the biologically active effect and stimulates the innate immunity [15,16]. They are generally recognized as safe by the US Food and Drug Administration and by Directive 295/77 of the Official Journal of the European Union (CELEX-2017); β–glucans from yeast are authorized for consumption as food supplements in concentrations of up to 1.275 g/day for people over 12 years old and in the adult population, and 0.675 g/day for children under 12 years old. These compounds isolated from yeast *Saccharomyces cerevisiae* are insoluble in water but are dispersible in many liquid matrices [17].

In terms of diabetes, yeast β–glucans have a certaindirection of action and are especially related to the innate immunity by activating several β–glucan receptors in macrophages. Therefore, the oral administration of β–glucan in mice promotes the homeostasis of glucose and lipids in the liver, discoveries with potential in diabetes and obesity [18]. Studies conducted in 2014 concluded that by stimulating the innate immune system via the Dectin-1 signaling pathway, β–glucans might be exploited by modulating the action of T cells responsible for destruction of insulin-producing pancreatic β–cells, and thus it can ensure a long-term protection of pancreatic β–cells in diabetes type I [19]. Another study on 52 patients evaluates the potential of supplementation of vitamin D with yeast β–glucan in diabetic retinopathy, a microvascular condition of the retina due to complications from diabetes, and showed significant decreases in the C-reactive protein and leptin levels [20].

Pomegranate (*Punica granatum* L.) is one of the oldest fruits known to exert a strong antidiabetic effect [21,22]. The most important product is juice that represents 78% of the edible part of the fruit (52% of the total fruit) [23]. Pomegranate contains a wide range of phytochemicals including polyphenols, anthocyanidins, tannic acid, gallic acid and ellagic acid, whereby it exerts antioxidant activity and helps to prevent oxidative stress [24]. Most of these pomegranate-derived compounds have a wide range of vasoprotective effects. D. Wang et al. (2018), in an analysis of the effect of various pomegranate compounds on the body, concluded by stating that improvement in vascular function is due to a combination of factors including a reduction in oxidative stress, lipid peroxidation, increased nitric oxide and decreased serum glucose levels, attenuated platelet aggregation and lowered blood pressure. The main compounds from pomegranate that provide vascular protection are represented by the presence of hydrolyzable tannins, especially ellagic acid and gallic acid [25].

Studies in people with type II diabetes have focused on the effect of pomegranate juice. It was shown that a consumption of 40 g/day, in addition to the above-mentioned effects, resulted in reductionsin oxidative stress, total cholesterol and LDL after 8 weeks [26]. These findings of the mechanism of action of the bioactive compounds provide evidence of the antidiabetic role of pomegranate. In fact, the ellagitannins contained in pomegranate might induce the activation of eNOS synthase (with a cardiovascular protection role) in the endothelial cells of human arteries [27]. In 2020, Guerrero-Solano et al. (2020) determined that pomegranate and its secondary metabolites can be considered in the treatment of inflammatory pain, nociceptive pain and neuropathic pain [28].

In research on the potential effect of pomegranate in metabolic syndrome, Medjakovicand Jungbauer (2013) stated that the assimilation of ellagic acid from pomegranate juice or pomegranate powder is almost similar. The release and elimination from the blood occurs within 6 h independently of the form of administration. What differs is the pharmacokinetics of the process which is delayed in the administration of pomegranate powder [29].

Taken together, by choosing the right composition for film formulation, the bioactive film consisting of β–glucan, sodium alginate, pomegranate juice and glycerin, it becomes suitable in diabetes for packaging dry powdered pharmaceuticals. The first step of this study was to find the most suitable bioactive compounds for the formulation of films that show a positive effect on diabetes complications. The second step of this work was to develop and evaluate the interaction between β–glucan, sodium alginate and pomegranate juice. A response surface methodology (RSM) was performed to optimize the physical and barrier properties of the films. In the third step, after optimization using the Box-Behnken design and predicting an optimal sample, the β–glucan and pomegranate juice based film obtained experimentally was validated by resuming experiments andassessing the properties of the film. Additional tests were performed on the optimal sample such as: microbiological, UV-barrier properties and film microstructure. These investigations are required to ensure the microbiological quality of the film, UV protection of the packaged product and evaluation of whether is a risk of cracks developing.

The addition of sodium alginate was performed to form films with good mechanical resistance, cohesiveness and stiffness [30], while the presence of plasticizer-like glycerin is necessary to increase the elasticity and flexibility of the film [31].

## 2. Materials and Methods

### 2.1. Materials

Pomegranate juice (BioAgros, Pella, Greece) was purchased from a local supermarket. It is a clear juice extracted from pomegranate seeds by removing the outer and inner skin, without the addition of preservatives, additives or sugar. The dry matter of pomegranate juice determined was 12.42% *w/v* with a pH = 3.03. β–glucans were extracted from spent brewer’s yeast provided by the SC, Bermas SA brewery (Suceava, Romania). The other film components were: Sodium alginate, Product No. 9180.1 (Carl Roth, Karlsruhe, Germany) and Glycerin, Product No. G7893 (Sigma-Aldrich, St. Louis, MO, USA, ACS reagent ≥ 99.5%).

#### 2.1.1. Preparation of Yeast β–Glucan

Numerous studies demonstrated that β–glucan from spent brewer’s yeast has a better pattern to stimulate the immune system than baker’s yeast [32,33,34]. Thus, in order to extract β–glucans, the raw material was initially subjected to a purification process to remove residual products, to a debittering protocol to solubilize the bitter compounds adsorbed on the surface of the cell wall and finely to an alkaline-acid treatment to extract β–glucans.

The debittering process conductedaccording to [35] at 50 °C, pH = 10 with NaOH 2 N, 10 min to remove humulones and isohumulones. Next, yeast slurry was subjected to autolysis at 55 °C/24 h followed by an enzymes inactivation at 80 °C/15 min [36]. The alkaline-acid extraction is one of the most commonly used and safest extraction methods to obtain yeast β–glucans [37]. It was carried out according to [38] with the use of a NaOH 1.5 N solution in a ratio of 1:5 (*w*/*v*) (autolysed yeast cells to NaOH) heated at 90 °C/2 h under continuous stirring. After cooling, centrifugation and washing three times with distilled water were conducted; the pellet was treated with HCl 1 N in the same ratio (1:5 *w*/*v*) at 75 °C/2 h. This process was achieved in order to adjust the pH values and to remove the amorphous structures such as glycogen and mannans [39]. The wet β–glucan was centrifuged, homogenized and stored at 4 °C until used. An aliquot of β–glucan was taken to determine the moisture content necessary for the film formulation (17.32% *w*/*w*) and for FT-IR analysis.

#### 2.1.2. Preparation of Film-Forming Solution (FFS) and Casting

The film-forming solution (FFS) and casting were prepared according to the following method. Firstly, wet β–glucan with a known dry matter content (17.32% *w*/*w*) was added in each beaker for an equivalent of 1.5, 1 and 0.5 g dry matter of β–glucan (e.g., 8.66 g wet × 0.1732 = 1.5 g dry weight β–glucan). Then, sodium alginate was added in amounts ranging from 0.2 to 0.6 g. Pomegranate juice with a dry matter content of 12.42% (*w*/*v*) was used as 10, 20 and 30 mL according to the recommendations for daily use. Glycerin was added as a plasticizer in a proportion of 25% of the total weight of the solids. Each sample was solubilized in a total of 150 mL solvent (distilled water and the amount of pomegranate juice). Fifteen films were formulated according to a Box-Behnken design based on RSM. The heat treatment applied was 80 °C for 30 min under continuous stirring at 900 rpm to form a homogenous solution. Then, 15 min before the end of the process, the pomegranate juice was added into the beakers for a slight sterilization and for incorporation into the FFS.

These mixtures were then casted into 8.6 cm diameter Petri dishes. A total of 30 mL of each FFS were dispersed on Petri dishes and then dried at 40 °C in a ventilated oven for 48 h. The dried films were stored at room temperature (24 °C) and a relative humidity of 31% RH until the analysis.

### 2.2. Methods

#### 2.2.1. Thickness and Density Measurements

A PosiTector 6000 (DeFelsko, Ogdensburg, NY, USA) thickness gauge with a high resolution and accuracy (0.1 µm in a range of 0.0–999.9 µm) was used to determine the thickness of the films. The measurements were taken at ten different points, and the mean was used in the calculation of film properties. Film thickness was expressed in µm.

Density was determined by dividing the weight of each film, cut into a square of 3 cm × 3 cm (with its known thickness) by volume of the film. Density of the films was calculated as follows:

$$\mathrm{Density}=\frac{W}{V}\times 10^{4}\;(g\times {\mathrm{cm}}^{-3})$$

were *W* is the weight of the film of a square of 3 cm × 3 cm (g), and *V* is the volume of film (3 cm × 3 cm × film thickness µm); 10^4^ is the conversion factor from µm to cm of the film thickness.

#### 2.2.2. Water Vapor Transmission Rate (WVTR)

The water vapor transmission rate (WVTR), or the rate of water vapor permeating through the film, is based on the diffusion of water vapor from a higher partial pressure of water vapor to a lower partial pressure [40]. The WVTR tests were measured using the dry cup method according to the ASTM E96/96M method [41]. When the desiccant method was performed, circular pieces of films were sealed horizontally on polystyrene Petri dishes containing approximately 10 g of CaCl_2_ as the desiccant. The sealant chosen in the experiment was highly resistant to the water vapor and did not gain weight over the required period of time. The air gap between the CaCl_2_ and films was about 4 mm. The cups were placed in an environmental chamber with a saturated solution of NaCl at the bottom providing a 75% RH. The WVTR of the films was calculated by dividing the slope (the weight gain versus time) to the area of exposed film using the following equation:

$$\mathrm{WVTR}=\frac{S}{A}\;(g\times h^{-1}\times m^{2})$$

where *S* is the slope or the amount of water gain in unit of time (ΔW/Δt in g×h^−1^), and *A* is the area exposed to water diffusion transfer (m^2^).

The weight of the cups was measured every 8 h within 72 h. We record ten data points over the 72 h time to provide accurate information about the amount of water gain. The regression coefficients of the slope were greater than 0.9923 which indicates high accuracy in weighing and handling samples.

#### 2.2.3. Water Vapor Permeability (WVP)

Water vapor flux through the film, or WVP, is a parameter that describes how easily a film will be penetrated by water vapor [42]. The water vapor permeability (g×mm×kPa^−1^×h^−1^×m^−2^) of the films was determined by dividing the WVTR to water vapor partial pressure across the film and multiplying by the thickness of the film [43]. The WVP is in accordance with the following equation:

$$\mathrm{WVP}=\frac{\mathrm{WVTR}\times L}{\Delta p}=\frac{S}{A}\times \frac{L}{\Delta p}=\frac{\Delta w}{\Delta t\times A}\times \frac{L}{\Delta p}\text{→}(g\times \mathrm{mm}\times {\mathrm{kPa}}^{-1}\times h^{-1}\times m^{2})$$

where WVTR is the water vapor transmission rate (g×h^−1^×m^−2^); *L* is the thickness of the film (mm), and Δ*p* is the differential water vapor partial pressure across the film (kPa), which is calculated as follows [44]:

$$\Delta p=S\times \left(R_{1}-R_{2}\right)\;(\mathrm{kPa})$$

where *S* is the saturated vapor pressure of water (3.1687 kPa at 25 °C [45]); *R*_1_ and *R*_2_ are the moisture gradient (0.75 and 0, respectively).

#### 2.2.4. Moisture Content (MC) and Solubility

Moisture content was determined gravimetrically by using 3 cm × 3 cm samples from density measurements and drying them in an oven at 105 ± 1 °C for 24 h. The difference in the sample weight loss was determined, and MC was reported as the percentage of water removed as follows:

$$MC=\frac{W_{0}-W_{1}}{W_{0}}\times 100\;(\%)$$

where *W*_0_ is the initial weight of the sample, and *W*_1_ is the final weight of the dried film.

The solubility of the films is an indicator of the strength of the packaging when exposed to a water-containing environment [46]. Potential applications often involve water insolubility to improve integrity and water resistance, but in some cases increased solubility is desirable, as is the casewith fast dissolving films. For this, 2 cm c2 cm square strips from each sample were cut and immersed in a volume of 50 mL of distilled water. The samples were vigorously shaken until dissolved. The dissolution time (minutes) was recorded using a chronometer.

#### 2.2.5. Film Opacity and Color Measurement

Besides the important role in consumer acceptability, the opacity of the films gives information about the homogeneity of the particles dispersed in film and for any pores formed [47]. A high opacity of the films could indicate an increased number of pores filled with dispersed ingredients which reduces the light pathway. The opacity of each sample (cut in rectangular 1 cm × 4 cm pieces) was obtained at 600 nm by dividing the absorbance to the film thickness described previously by [48] and follows the equation:

$$\mathrm{Opacity}=Abs_{600nm}/L\;(A\times {\mathrm{mm}}^{-1})$$

where $Abs_{600nm}$ is the absorbance at 600 nm, and *L* is the film thickness (mm).

The absorbance was read on an UV-VIS-NIR Shimadzu 3600 spectrophotometer (Tokyo, Japan).

The color profile analysis of films, lightness (L), redness (a*), yellowness (b*) were measured using a chromameter CR-400 (Konica Minolta, Tokyo, Japan).

#### 2.2.6. Water Activity

Water activity (a_w_) is defined as the ratio of the partial pressure of water vapor in the product to that in the presence of pure water and is a measurement that gives information if the product has reached the critical point whether or not spoilage reactions can occur [49]. The water activity was measured with a water activity analyzer AquaLab 4TE (Meter Group, Inc., Pullman, WA, USA). The chilled-mirror dew point technology was applied by the instrument to determine a_w_ values with a resolution of 0.0001 in a range of 0.030–1.000.

#### 2.2.7. FT-IR Spectroscopy

Fourier transform infrared spectroscopy (FT-IR) with attenuated total reflectance unit (ATR) analysis was performed using a Nicolet iS-20 FT-IR Spectrometer (Thermo Scientific, Karlsruhe, Dieselstraße, Germany). ATR-FT-IR measurements were achieved by placing samples directly onto the ZnSe ATR crystal plate. The spectra were collected over a range of 4000–650 cm^−1^ with 32 scans per spectrum at a resolution of 4 cm^−1^.

#### 2.2.8. Scanning Electron Microscopy (SEM)

The microstructure and cross-section of films were achieved using a scanning electron microscope VEGA II LMU (Tescan, Brno, Czech Republic) under HighVac conditions by using a secondary electron (SE) detector operated at an accelerating voltage of 30 kV and a magnification of 1 and 1.5 kx without preliminary coatings on the investigated surface.

#### 2.2.9. Model Fitting

For the optimal design of the experimental model, a Box-Behnken factorial model was generated in which a total of 15 experiments were performed with three different levels of each constituent: β–glucan (0.5, 1, 1.5 g), sodium alginate (0.2, 0.4, 0.6 g) and pomegranate juice (10, 20, 30 mL), with 3 central points as shown in Table 1 and Table 2. A summary of the experimental design is shown in Table 2 and is based on the interval set for each variable.

## 3. Results and Discussion

### 3.1. Optimization of Film Forming Solution by Response Surface Methodology

#### 3.1.1. Statistical Design

All experiments were performed according to the experimental design showed in Table 1 which presents the coded and uncoded values for each of the variables.

The design of experiment software (DOE) (Design Expert, trial version, Stat-Ease, Inc., Minneapolis, MN, USA) suggested 15 model runs with three center points and the response values of each run determined through the experiment series. Table 2 shows the experimental and coded values of the 15 runs determined by the model, and Table 3 lists the composition of each film.

In this design, the relationship of the coded variables β–glucan (*X*_1_), sodium alginate (*X*_2_) and pomegranate juice (*X*_3_) were determined by fitting the second order polynomial equation to the data obtained. The model proposed for the response *Y* is given below:

$$Y=b_{0}+b_{1}X_{1}+b_{2}X_{2}+b_{3}X_{3}+b_{4}X_{1}X_{2}+b_{5}X_{1}X_{3}+b_{6}X_{2}X_{3}+b_{7}X_{1}^{2}+b_{8}X_{2}^{2}+b_{9}X_{3}^{2}$$

where *Y* is the estimated response; *X*_1_ is the β–glucan content (0.5, 1, 1.5 g); *X*_2_ is the sodium alginate (0.2, 0.4, 0.6 g); *X*_3_ is the pomegranate juice (10, 20, 30 mL); *b*_0_ is the constant of the equation; *b*_1_, *b*_2_, *b*_3_are the coefficients of the linear terms; *b*_4_, *b*_5_, *b*_6_ are the coefficients of the interaction effects, and *b*_7_, *b*_8_, *b*_9_ are the coefficients of the quadratic effects.

The coefficient of determination (R^2^) was used to verify if the polynomial model is adequate. *F* and *p* values were computed for all responses. The software was used to evaluate the results of the quadratic polynomial model.

Numbers in brackets are the dry matter of β–glucan reported to the moisture content of 17.32% *w*/*w* (0.5, 1, 1.5 g, respectively).

To calculate the amount of 25% (*w*/*w*) glycerin required for each film-forming solution (FFS), the total dry matter (DM) of each compound was considered. Thus, for example, following calculation, the amount of glycerin for sample 0.5-0.4-30 with 0.5 g β–glucan, 0.4 g sodium alginate and 30 mL pomegranate juice (PJ) was determined to be 1.16 g ((2.89 g wet β–glucan×0.1732 DM)+(0.4 g sodium alginate)+(30 mL PJ×0.1242 DM))×0.25=1.16 g Glycerin.

#### 3.1.2. Response Surface Analysis

A clear image of the experimental responses for β–glucan, sodium alginate, pomegranate juice, and glycerin films is revealed in Table 4. The regression coefficients of the quadratic model for each response as well as the significance of the regression (*F*-values), *p*-values and the coefficient of determination (R^2^) are shown in Table 5.

- Thickness and density

The experimental results showed that the thickness and density of the films are dependent on factors such as the amount of β–glucan and sodium alginate (SA) and notably on the volume of pomegranate juice (PJ) which has an important impact on the layer thickness. The highest value of thickness was found in sample 1-0.6-30 with 147.30 µm with a content of 1 g β–glucan, 0.6 g sodium alginate and 30 mL pomegranate juice. On the other hand, the lowest thickness was found in sample 0.5-0.4-10 with 64.40 µm having 0.5 g β–glucan, 0.4 g SA and 10 mL PJ.

**Figure 1:**
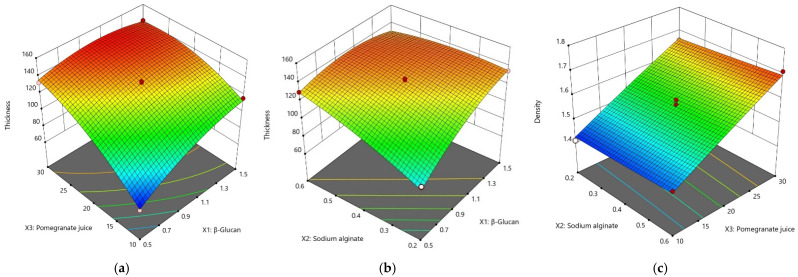
3-D plots for the effect of independent variables on the film thickness and density. (**a**) Effect of β–glucan and PJ on the film thickness; (**b**) Effect of SA and β–glucan on the film thickness; (**c**) The effect of SA and PJ on density of films.

Following the second order polynomial equation, it was found that thickness has a quadratic relationship with the three variables β–glucan (X_1_), sodium alginate (X_2_) and pomegranate juice (X_3_). Thus, the Thickness = 133.13 + 13.11X_1_ + 10.12X_2_ + 24.01X_3_ − 12.15X_1_X_2_ − 10.82X_1_X_3_ − 7.55X_2_X_3_ − 8.75X_1_^2^ – 4.28X_2_^2^ − 10.7X_3_^2^; R^2^ = 0.9912; *p* < 0.05; *F*-value = 62.28 (Table 5). From the above equation, we can observe that thickness is directly proportional to the amount of β–glucan, SA and PJ added to the film-forming solution. However, we can observe that the effect of PJ on the film thickness is observed to be the most significant (+24.01X_3_). Hence, if we want to obtain a thin film, the first parameter to be reduced is pomegranate juice (X_3_) and then the other two factors (Figure 1a,b). The value of R^2^ of 0.9912 indicates that 99.12% of the total variation is relevant to the model. The *F* value of the 62.28 model implies that the model is significant, and there is only a 0.01% chance of being affected. The highest thickness values of β–glucan films were approximately 150 μm found by Sherafatkhah et al. (2021) [50] and 120 μm made from baker’s yeast β–glucan and glycerin [51].

Regarding density, a high-density film provides a compact structure and can be attributed to better water barrier properties [48]. Of course, low-density films could be correlated with high flexibility. Films such as LDPE/starch blends for food packaging with varying densities from 0.921 to 1.195 g/cm^3^ will have significantly higher tensile and elongation strength at 0.921 g/cm^3^ (10.78 MPa and 52%, respectively) compared to densities of 1.195 g/cm^3^ (only 3.92 MPa and 8%) [52]. The best model equation for density was Density = 1.56 + 0.005X_1_ + 0.01X_2_ + 0.12X_3_ − 0.01X_1_X_2_ + 0.005X_1_X_3_ + 0.002X_2_X_3_ + 0.003X_1_^2^ + 0.001X_2_^2^ − 0.003X_3_^2^; R^2^ = 0.9473; *p* < 0.05; *F*-value = 9.98. A high-density film is correlated with the amount of PJ added to the film composition (Figure 1c). The R^2^ value indicates that the regression model explains a total variation of 94.73%, while the *p* value of 0.0104 implies that the model is significant.

The density of the films varies between 1.41 and 1.71 g/cm^3^;the lowest density is found in film with 1 g of β–glucan, 0.2 g of SA, 10 mL of PJ, and the highest density is found in the film consisting of 1 g β–glucan, 0.6 g SA, 30 mL PJ.

- Water vapor transmission rate (WVTR)

Along with water vapor permeability, WVTR is one of the most important studied properties of films due to its role in food spoilage reactions [53]. The amount of water transferred through the film in the packaged product is recommended to be as small as possible to reduce the amount of moisture uptake of the products.The water vapor transmission rate of the film was calculated from the slope in a unit of time. The model equation for WVTR showed a quadratic relationship of the form:

WVTR = 28.17 − 1.02X_1_ + 1.07X_2_ + 3.1X_3_ − 0.23X_1_X_2_ − 0.20X_1_X_3_ − 0.10X_2_X_3_ + 0.86X_1_^2^ – 0.64X_2_^2^ − 1.2X_3_^2^; R^2^ = 0.9824; *p* < 0.05; *F*-value = 30.92

As can be seen, the value of R^2^ indicates that the regression of the model can explain 98.24% of the total variation. A low value of *p* of 0.0007 indicates that the terms of the model are significant (*p* < 0.05). Thus, from the equation of the model for WVTR, we noticed that the most significant increase in the water vapor transmission rate through the films occurs due to a high level of pomegranate juice (3.1X_3_) and is followed by the content of sodium alginate. In contrast, the WVTR value varies inversely with β–glucan content (−1.02X_1_).

WVTR values range from 22.3472 g×h^−1^×m^−2^ to 32.1869 g×h^−1^×m^−2^. The highest value is found in sample 0.5-0.4-30 with 30 mL of PJ, 0.4 g SA and 0.5 g β–glucans. On the other hand, the lowest value is observed in the film 1-0.2-10, which contains 1 g β–glucans, 0.2 g SA and 10 mL PJ. These results are much lower than those found by Costa et al. (2020), who developed films containing pomegranate extract, polyvinyl alcohol and starch. The data found by them are between 1415 and 1601 g×day^−1^×m^−2^ (between 58.95 and 66.70 g× h^−1^×m^−2^, double than our results obtained) [54]. Besides different proportions of the ingredients, one of the reasons why the results showed a wide variation is that films based on starch and PVA are more susceptible to the water vapor flux [52]. Another explanation is that β–glucans create more hydrophobic films and decreased the WVTR values.

- Water vapor permeability (WVP)

Water vapor permeability (WVP) tests obtained by multiplying the WVTR by the film thickness (in mm) and dividing the partial pressure of water vapor are shown in Table 4. Films made of β–glucan, SA, PJ and glycerin showed significantly lower WVP values. The highest value of WVP, 1.8657 g×mm×kPa^−1^×h^−1^×m^−2^, was found in films with 1 g β–glucan, 0.6 g SA, 30 mL PJ and has a thickness of 147.3 µm. The lowest value of the water vapor flow across the film of 0.6780 g×mm×kPa^−1^×h^−1^×m^−2^ was found in sample with 10 mL PJ, 0.4 g SA and 0.5 g β–glucan.

The present study found that WVP fits a quadratic regression model as:

WVP = 1.58 + 0.09X_1_ + 0.16X_2_ + 0.42X_3_ − 0.15X_1_X_2_ − 0.12X_1_X_3_ − 0.06X_2_X_3_ − 0.06X_1_^2^ − 0.07X_2_^2^ − 0.15X_3_^2^; R^2^ = 0.9950; *p* < 0.05; *F*-value = 109.58

**Figure 2:**
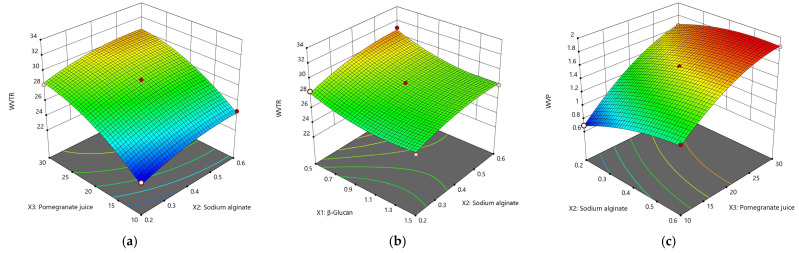
3-D surface plots for the effect of independent variables on the film WVTR and WVP. (**a**) Effect of SA and PJ on the rate of water vapor permeating through the film (WVTR); (**b**) Effect of SA and β–glucan content on WVTR; (**c**) The effect of SA and PJ on the WVP.

The WVP model is suitable for predicting variation (R^2^ = 0.9950), and a *p*-value < 0.05 indicates that the data fitting was significant. Thus, an increase in WVP values is directly proportional to the amount of pomegranate juice, sodium alginate and to a small extent with the added β–glucans (0.09X_1_). When a low value of water vapor flow across the film is desirable, pomegranate juice and sodium alginate should be reduced (Figure 2c).

In addition to the total dry matter, two other explanations for WVP support the data obtained. One of these is the thickness, strongly correlated with WVP (R^2^ = 0.958); thin films will present low values of WVP (0.6780 g×mm×kPa^−1^×h^−1^×m^−2^ at 64.4 µm). Secondly, due to a strong hygroscopic character, a large amount of plasticizer will increase the WVP values, and from Table 3 we can observe that only 0.54 g were added to the film with the lowest WVP value (sample 0.5-0.4-10), while 1.33 g glycerin were added to the sample with the highest WVP level (1-0.6-30).

- Moisture content (MC)

Changes in moisture content (MC) in β–glucan films ranged from 12.31 to 18.49%. The experimental MC data were adapted to the quadratic equation (Table 5), and the analysis of the variance showed that the quadratic model is significant (*p* < 0.05) with a regression coefficient R^2^ = 0.9292. The amount of PJ and β–glucan (*p* < 0.05) has a significant influence on the moisture content. Thus, from the moisture equation MC = 13.96 − 1.09X_1_ − 0.72X_2_ + 1.05X_3_ + 0.85X_1_X_2_ + 1.51X_1_X_3_ + 0.54X_2_X_3_ + 1.48X_1_^2^ − 0.01X_2_^2^ + 1.15X_3_^2^; R^2^ = 0.9569; *p* < 0.05; *F*-value = 12.34, it can be seen that the moisture increases as PJ increases, and decreases with the addition of β–glucan (−1.09X_1_). The amount of sodium alginate does not have a significant influence on the quadratic model (*p* = 0.0624); thus, it cannot be considered for this model.

The lowest MC value of 12.31% was found inthe sample with 1.5 g β–glucan, 0.4 g SA and 10 mL PJ. Nabi and Jafar (2021) found that an increase in the glycerin concentration of the films will significantly increase the moisture content to values up to 32.86% [44]. A relationship between the addition of glycerin and moisture content can be observed in Table 3; the highest MC of 18.49% was the same with the highest amount of plasticizer of 1.41 g.

- Film solubility

**Figure 3:**
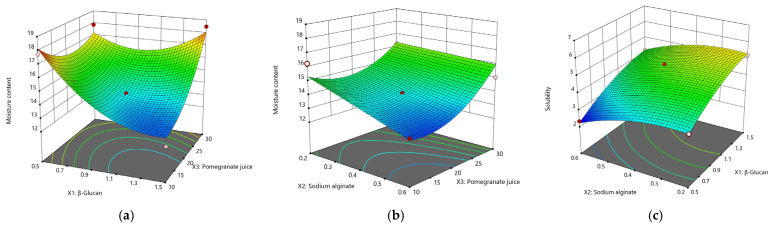
3-D surface plots for the effect of independent variables on the film moisture content and solubility. (**a**) Effect of β–glucan and PJ on MC; (**b**) Effect of SA and PJ on MC; (**c**) Effect of SA and β–glucan on the solubilization time.

In assessing the degree of solubility, the values varied between 2.34 min and 6.33 min, mainly compared to the amount of sodium alginate added and β–glucan content.From the following equation, Solubility = 4.56 + 0.97X_1_ − 0.57X_2_ + 0.23X_3_ + 0.01X_1_X_2_ − 0.88X_1_X_3_ + 0.06X_2_X_3_ − 0.34X_1_^2^ − 0.41X_2_^2^ + 0.17X_3_^2^; R_2_ = 0.9214; *p* = 0.0264; *F*-value = 6.51, it is observed that the level of sodium alginate has a direct effect on the solubility of the films; an increased amount of SA will reduce the solubilization time of the films (−0.57X_2_). β–glucan will slightly increase the film solubilization time (+0.97X_1_) while PJ (X_3_) is insignificant for the quadratic model (*p* = 0.2464). The shortest solubilization time was found in films with 0.5 g β–glucan, 0.6 g SA and 20 mL of PJ with 2.34 min, while the longest dissolution time of 6.33 min was found inthe sample with 1.5 g β–glucan, 0.4 g SA and 10 mL PJ. Figure 3a–c shows the 3-D evolution of the moisture and solubility content of the films according to the three parameters involved in optimization.

- Opacity and color

The opacity and film color are two important optical properties that are determined by the scattering of light, mainly due to the concentration and particles in the biopolymer and, respectively, due to the selective absorption of light waves [55]. Besides the consumer acceptability, the opacity of the films has a direct impact on the appearance of the coated products. A high degree of transparency is better, but an increase in opacity helps to enhanceof the shelf life of packaged products [56].

Opacity was found to have a quadratic relationship with the three process variables of β–glucan content (0.5, 1 and 1.5 g), sodium alginate (0.2, 0.4 and 0.6 g) and pomegranate juice (10, 20 and 30 mL). The quadratic regression model (Table 5) is statistically significant (*p* < 0.05) with a regression coefficient R^2^ = 0.9569. The lowest opacity value of 1.46 was found in sample 0.5-0.6-20 with 0.5 g β–glucan, 0.6 g SA and 20 mL of PJ, while the highest value was found in sample 1-0.2-10 with 1 g β–glucan, 0.2 g SA and 10 mL PJ with an opacity value of 10.51.

The color profile of the films shown in Table 5: CIE L a*b* reflects the brightness characteristics of L (between 0 and 100), a* (positive for red or negative for green) and b* (positive for yellow and negative for blue). From the analysis of the variance of the quadratic model for brightness, redness and yellowness, it was observed that none of these responses matched a specific pattern. ANOVA for the model showed that the color profile in the format L, a*, b* is insignificant (*p* > 0.05) for parameter optimization.

The highest brightness level of 33.43 was found in film with a low content of PJ (10 mL), 1.5 g β–glucan and 0.4 g SA. The tendency to red +a* was observed in films with a high content of PJ, SA and β–glucan (0.6 in sample 1.5-0.6-20).The yellow +b* hue was also increased in samples with high PJ; the highest value of 2.38 was found in the sample with 30 mL of PJ, 1.5 g β–glucan and 0.4 g SA. Instead, a film with a chromagreen-blue (a* = −0.02, b* = 1.16) was found in the sample with 10 mL PJ, 0.2 g of SA and 1 g of β–glucan.

#### 3.1.3. Water Activity (a_w_)

The water activity profile (a_w_) for each of the films varies in a relatively small range as can be seen in Table 6. The water activity ranges between 0.2148 and 0.2745. There was no correlation between the composition of the films and the values of the water activity resulting in such a limited range.

It is important to note that this indicator is a measure of water availability for biological reactions. According to Beuchat (1983), there is a relationship between water activity and microbial growth; food spoilage starts at a_w_ 0.61–0.85 with the formation of mold and yeast, and over a_w_ 0.85 bacteria grows [57]. A water activity value less than 0.6 limits the growth of any microorganisms, so a required minimum water activity of 0.6 should still be relevant for films that will serve as packaging supplements/dry powdered pharmaceuticals.

#### 3.1.4. FT-IR Spectroscopy

**Figure 4:**
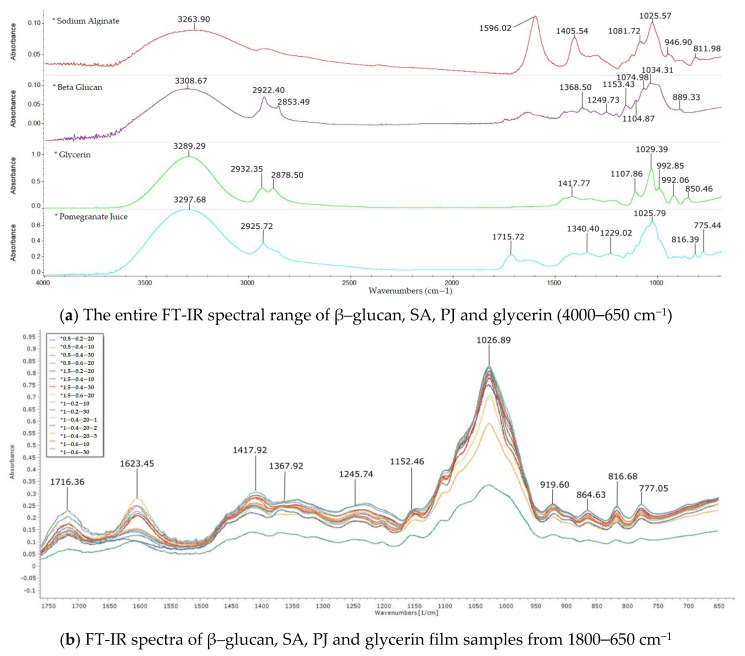
FT-IR spectra: (**a**) Spectra of β–glucan, sodium alginate, pomegranate juice and glycerin before the films were produced (4000–650 cm^−^^1^); (**b**)FT-IR spectra of the film samples (1800–650 cm^−^^1^).

FT-IR spectra near the wavenumbers of 1160, 1078, 1044, 890 cm^−1^ are characteristic for β–1,3/β–1,6-glucan from yeast [58]. The bands of the β–glucan aliquot using the ATR-FT-IR technique were 1249.73, 1153.43, 1104.87, 1074.98, 1034.31 and 889.33 cm^−1^, respectively (Figure 4a). The peaks found near the wavenumbers 1153.43 and 1104.87 cm^−1^ are characteristic for β–1,3-glucan configuration [59], while the wavenumbers 1074.90 and 889.32 cm^−1^ are specific for the β–1,6 configuration and β conformation found in yeast β–glucans [59,60].

The FT-IR spectra of the films made with different amounts of β–glucan, sodium alginate, pomegranate juice and glycerin showed close peaks in the fingerprint region such as those shown in Figure 4b. Thus, a shift in the absorption maxima towards a higher or lower wavenumber can be observed. Slightly bathochromic shifts were observed in all samples which was more likely related to the newly intermolecular bonds created with a noticeable shift from 889.33 cm^−1^ to 919.60 cm^−1^ characteristic for the β–glycosidic configuration. Moreover, the peak specific for β–1,3-glucan at 1152.46 cm^−1^ was found in all 15 analyzed samples. The addition of glycerin was observed by the increased O-H deformation band around 1417.92 cm^−1^ and sodium alginate at 1026.89 cm^−1^. The addition of pomegranate juice was visible in the absorbtion bands of 1716.36, 816.68, 777.05 cm^−1^ which occurs due to C=O stretching and O-H deformation of acids. Hence, we can conclude that the heat treatment applied did not affect the initial composition of the ingredients in such a manner as to lead to a degradation of the compounds in final film.

### 3.2. Optimization of Process Parameters for Optimal Film Development

**Figure 5:**
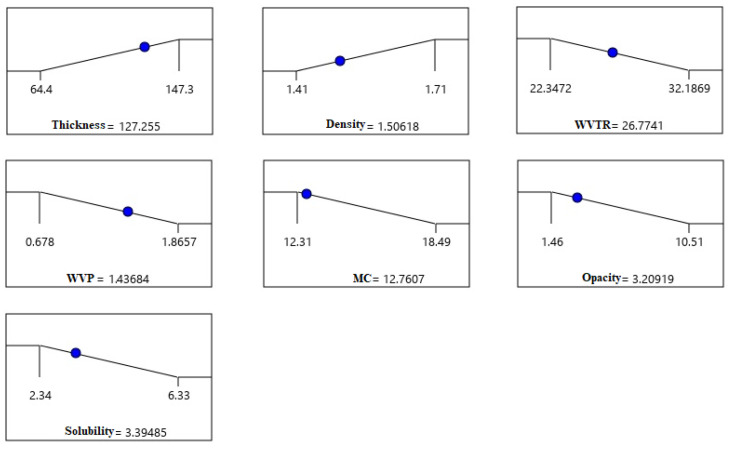
Desirability ramps for optimum points obtained with Design Expert software.

Each response was individually optimized using Design Expert. An optimum cannot be found at the stationary point where the slope of the response surface is reduced to zero in all directions. Therefore, for example, it is desirable that the transport of water vapor through the films to be as low as possible, and thus the WVTR and WVP will have to be small. A thick film, and at the same time transparent to allow the visible radiation to penetrate into the packaged product, is also desirable. Moreover, in order to ensure a faster dispersion of the packaged products into liquids, it is recommended that the developed packaged producthavea high solubility. The density of the film was also taken into account because a high density film also means a film with a much more compact structure and can be attributed to better water barrier properties. The color of the samples did not vary significantly to be considered and was left in range. The optimal values for each answer were constrained to intervals set by minimum or maximum as shown in Figure 5.

Through the optimization function, each response selected was adapted according to intervals obtained from the experimental data. The program found a total of 36 solutions from which the best film with the desired characteristics above and bringing a sufficient amount of β–glucan and PJ could be obtained by using 0.98 g of β–glucan, 0.6 g of SA and 14.80 mL of PJ.

For the independent variables, the optimal values estimated by the program will be: WVTR of 26.774 g×h^−1^×m^−2^, WVP of 1.437 g×mm×kPa^−1^×h^−1^×m^−2^, a film thickness of 127.25 µm, density of 1.506 g×cm^−3^, opacity of 3.2%, moisture content of 12.76%, a solubilization in 3.39 min and color values L = 32.62, a* = 0.18 and b* = 1.56.

#### 3.2.1. Validation and Confirmation of Computationally Predicted Data

To confirm the predicted data, a film with 1 g of β–glucan, 0.6 g of SA and 14 mL of PJ was developed (hereinafter referred to as sample 1-0.6-14). The amount of glycerin introduced in the composition of this film was 0.83 g (25% of the dry matter). Experimental recheking was performed on thickness, density, WVTR, WVP, opacity, solubility, moisture content, color and water activity.

In addition, the optimal samplewas evaluated over the entire ultraviolet-visible spectral range (200–800 nm) using the Shimadzu 3600 UV-VIS-NIR spectrophotometer (Tokyo, Japan). Microbiological tests were carried out to identify possible pathogens using Compact Dry plates, and finally film microstructure was evaluated using a scanning electron microscope (SEM).

**Figure 6:**
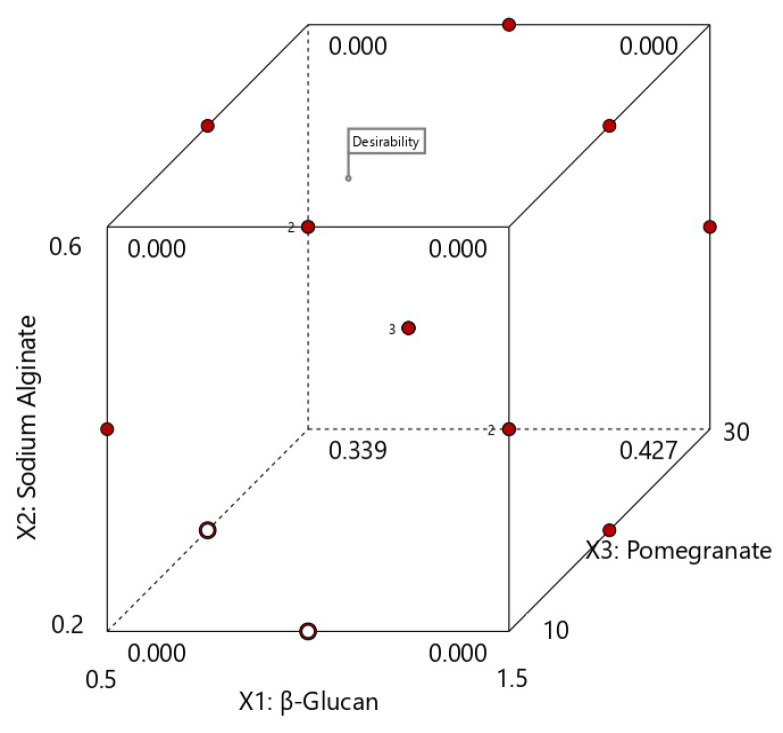
Results of the optimization process with the desirability of optimal for the three variables investigated.

By choosing the most appropriate predicted composition using the desirability function, experiments covering the desired mixtures were performed, and the predicted and experimental values were compared. As can be seen from Table 7, the experimental results are in a good correlation to predicted data with relative errors less than 5%. Therefore, the data (Figure 6) of the optimization process validated our experimantal results. The optimal results after performing the verification tests resulted in: WVTR of 27.1224 g×h^−1^×m^−2^, WVP of 1.390 g×mm×kPa^−1^×h^−1^×m^−2^, film thickness 121.4 µm, density 1.47 g×cm^−3^, opacity 4.48, moisture content 14.59%, solubilization time of 4.53 min and a color value for L = 36.45, a* = −0.05 and b* = 2.54. Moreover, the water activity determined at 25.06 °C was 0.2287 and is below the optimal microbial growth limit.

Based on the results, we can state that the DOE software can be proposed as befitting for films’ development in the optimizing parameters such as thickness, density, water vapor transmission rate, water vapor permeability, moisture content, solubility, opacity and color.

#### 3.2.2. Evaluation of the Optical Properties on Optimal Film in the UV-VIS Region

**Figure 7:**
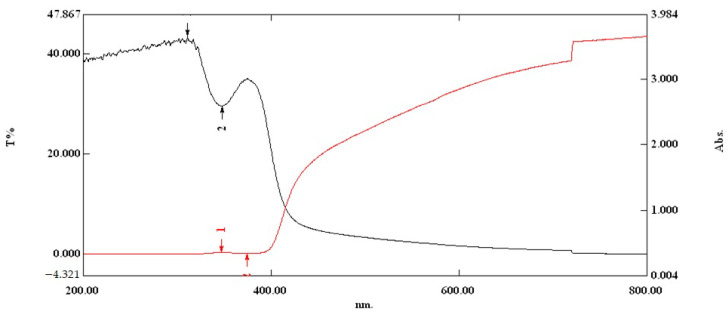
The absorption and transmission spectra of the optimal sample (200–800 nm).

Beside the opacity of films, which are determined experimentally by dividing the absorbance found at the wavelength of 600 nm to the film thickness in mm, an evaluation of the optical properties of the film over a wide spectral range is essential especially in the UV-region because the film must ensure a good protection of the packaged product. These products includes vitamins, lipids or edible oils that are sensitive to the UV radiation [42]. The ultraviolet range is between 100 and 400 nm while the visible spectrum is between 400 and 700 nm. By using the Shimadzu 3600 UV-VIS-NIR spectrophotometer (Tokyo, Japan), a scan was performed on a range between 200–800 nm to observe the light transmission through the optimal sample. Figure 7 shows the light transmission (red line and indicated with 1) passing through the film, and with 2 the light absorption in the range of 200–800 nm (black line). As can be seen, the transmission in the 200–400 nm is 0, a sign that ultraviolet radiation is blocked by the film containing β–glucan, sodium alginate, pomegranate juice and glycerin. As the wavelength increases from 400 to 800 nm, the light transmission in the visible region becomes exponential. At the same time, film absorbs the UV radiation between 200–400 nm, and from 400–800 nm the visible light passes through the film.

#### 3.2.3. Microbiological Analysis of Optimal Sample

This study was carried out in order to ensure the microbiological quality of the optimal film and thus implicitly will provide a guarantee on the packaged product. For this analysis, chromogenic ready-to-use Compact Dry plates (NISSUI pharma, Tokyo, Japan), suitable for controls during the preparation or use of the finished products, were used, as well as the films with β–glucan, SA, PJ and glycerin. Regarding the pathogens commonly found in packaged products, the following were selected for analysis: total number of aerobic mesophilic bacteria (TC) that provides a quantitative estimation of the concentration of microorganisms such as bacteria in a sample; coliform bacteria (CF) defined as Gram-negative bacteria and responsible for mild diseases that may worsen depending on the pathogen; EC chromogenic plates specific for 3 types of coliform bacteria such as *E. coli* (blue colonies), *E. aerogenes* (red colonies), *P. aeruginosa* (brown colonies); XSA—specific for *Staphylococcus aureus*, Gram-positive bacteria that can lead to sepsis; SL—for *Salmonella* and ETC for *Enterococcus* that can occur in the contamination with a variety of fermented foods.

**Figure 8:**
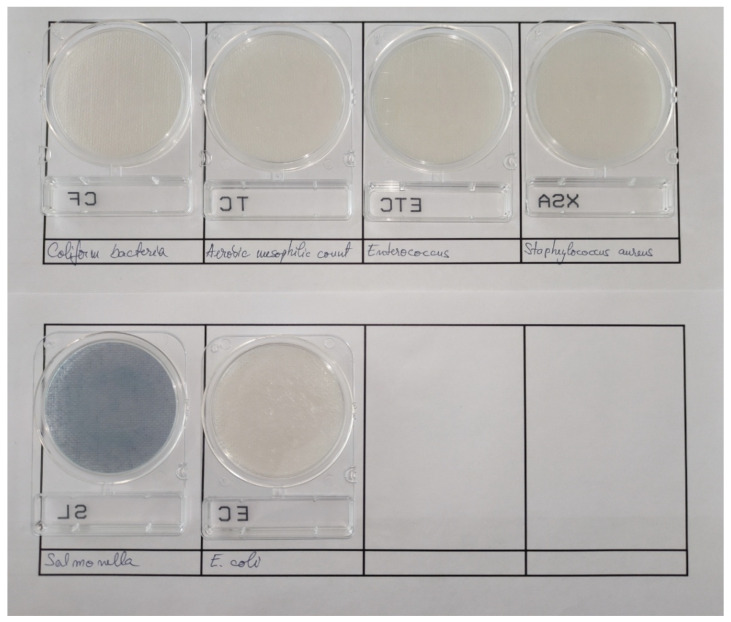
Results of microbiological tests for the optimal sample. No visible colonies were detected.

Sample preparation was performed by dissolving the film in distilled and sterilized water according to the AOAC standards described for each selective media. From the dissolved sample, 1 mL was inoculated into each plate containing specific nutrients. After inoculation, samples were incubated at 37 °C/24 h (for EC, CF, ETC), 37 °C/48 h for the total number of aerobic mesophilic bacteria (TC) and 41 °C/24 h for *Salmonella*. The results of the microbiological tests are shown in Figure 8.

According to the results, no microorganisms such as coliform bacteria, mesophilic aerobic bacteria, *S. aureus*, *E. coli*, *Salmonella* or *Enterococcus* grew on selective culture media. Of course, the results are supported by the strong antimicrobial activity of yeast β–glucans against two Gram-positive bacteria (*B. cereus* and *S. aureus*) and for three types of Gram-negative bacteria (*E. coli, Salmonella, P. vulgaris*) [61]. Pomegranate juice also exerts antibacterial activity on a wide range of bacteria such as *Salmonella* and is thought to have a stronger inhibitory effect than tetracycline against *Pseudomonas aeruginosa* [62].

#### 3.2.4. Scanning Electron Microscopy (SEM)

**Figure 9:**
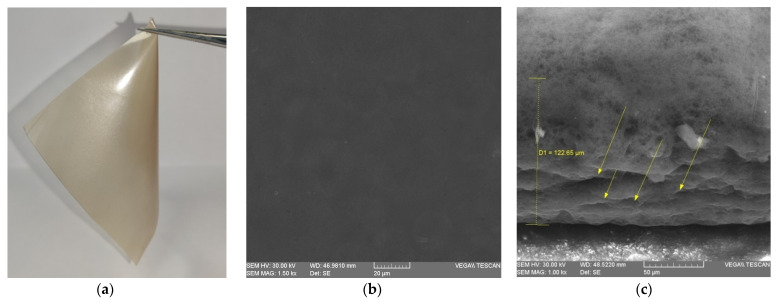
Optimal film and SEM micrographs. (**a**) Film made of 1 g β–glucan, 0.6 g SA, 14 mL PJ and 0.83 g glycerin; (**b**) Upper view SEM micrographs (1.5 kx); (**c**) Cross section micrographs of the film (1 kx).

SEM imaging was used to observe the film microstructure of the optimal film made with 1 g β–glucan, 0.6 g SA, 14 mL PJ and 0.83 g glycerin. Following the SEM analysis, the obtained film (Figure 9a) was subjected to an acceleration voltage of 30 kV at two different angles. A scan was performed perpendicular to the film (Figure 9b) to identify if there were any perforations or micropores in the structure and a cross section scan (Figure 9c) to identify possible microcracks.

While the top surface (Figure 9b) was a continuous structure without perforation points, micropores or microcracks, the cross section of the film (Figure 9c) presented longitudinal layers associated with drying and some discontinuities which are most likely cracks due to cutting of the film. In both optical and SEM images (Figure 9a–c), there can be observed a uniform distribution in the film structure without the existence of conglomerates. Moreover, the results confirm that a thickness of about 120 µm provides uniformity in the film, and, in the absence of pores, the membrane is more effective as a barrier against water vapor. The parallel longitudinal cracks (yellow arrows) in the cross section in the absence of a plasticizer can initiate, spread and lead to the film rupture. Therefore, the use of 0.83 g glycerin in the optimal film is recommended. Garcia et al. (1999), through a study on the microstructure and water vapor of films and coatings, concluded that, without a plasticizer, the presence of pores and cracks are obvious and increased the WVP values compared to those with a plasticizer in composition [63]. These imperfections and micropores found in films without a plasticizer will lead in propagation and film fracture [64].

### 3.3. The Packaging Concept of Dry Powdered Pharmaceuticals for Special Medical Purposes

Due to the hydrophilic nature of sodium alginate, the developed films can be easily sealed by wetting the edges with water. Based on the experimental results, the optimal film with a content of 1 g β–glucan, 0.6 g SA, 14 mL PJ and 0.83 g glycerin was used as packaging for powdered medical products for oral solution. The powder product was placed in the center of the film, and then the film was sealed without allowing the powder to come in contact with the wet edges.

**Figure 10:**
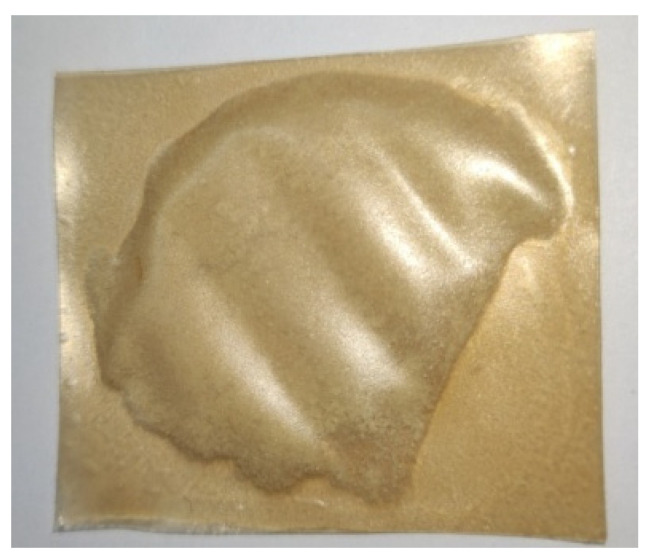
Application of β–glucanfilm with pomegranate juice for packaging powdered Vitamin C.

The high flexibility of the film allows obtaining packaging in different forms. Thus, Figure 10 shows a rectangular shape of packaging with vitamin C introduced by overlapping two films. Of course, bi-component packaging or that made by bending a single film can be produced.

## 4. Conclusions

The high costs of medication for chronic diseases give way to the use of alternative therapies such as dietary supplements or functional foods. Films based on β–glucan, sodium alginate, pomegranate juice and glycerin have been developed for use by people with diabetes and are of particular importance in preventing theassociated complications.

The method using the response surface of the Box-Behnken design was proved to be effective in optimizing the development process of these films. The dosage of the ingredients was chosen so that the consumption of β–glucan and pomegranate juice should be part of the daily diet of people with special needs and to contribute to a balanced diet. The three factor, three coded level factorial RSM was successfully used to optimize the effect of process variables such as thickness, density, WVTR, WVP, moisture content, solubility and opacity. The ideal film in response to the selected optimal values was developed using 1 g β–glucan, 0.6 g sodium alginate, 14 mL pomegranate juice, 0.83 g glycerin and distilled water up to 150 mL.

The experimental values of the optimal film resulted in: a minimum film vapor transfer rate (WVTR) of 27.1224 g×h^−1^×m^−2^, vapor permeability of water (WVP) of 1390 g×mm×kPa^−1^×h^−1^×m^−2^, a thickness of 121.4 µm, a water activity of 0.2287, a dissolution time of 4.53 min and a moisture content of 14.59%. A low amount of water passing through the film, important for the increase in the packaged product shelf life, was mainly associated with increased levels of β–glucans in the film composition and a low level of pomegranate juice and sodium alginate. The flow of water vapor through the film (WVP) is directly proportional to the WVTR and the thickness of the films. The determined moisture content of 14.59% provides indication that the film has a hydrophilic character, and the dissolution time of 4.53 min makes it optimal in applications for packaging water-soluble products for oral administration. Moreover, the water activity of 0.2287 falls within the limits in which there can be no reactions of microbial development which will provide stability to the packaged product.

FTIR spectroscopy showed the presence of all the compounds found individually in the films made; thus, no degradation of the compounds in the film such as β–glucans or pomegranate juice was identified. The transmission of the ultraviolet radiation in the range of 200–400 nm through the optimal film was zero, and thus the film becomes effective for the packaging of supplements or drugs requiring sunlight protection. Microbiological evaluation, in addition to water activity, suggests that no microbial development was observed. Moreover, for people with chronic disease such as diabetes, the film obtained must be free of pathogens to avoid the potential microbiological risks to the health of consumers. In fact, the chosen composition has a strong antifungal and antimicrobial effect. SEM microscopy on the flat surface of the film revealed a continuous structure, without pores, microcracks or perforation points. Longitudinal layers associated with drying were identified on the cross section of the film as well as some discontinuities that are most likely associated with the cutting-off of the film. The presence of the plasticizer in its composition will not allow the initiation of these longitudinal microcracks.

Tests such as the water vapor transmission rate, water vapor permeability or water activity were carried out at room temperature according to the standard test methods. Therefore, the packaging made from film based on β–glucan and pomegranate juice can be stored under ambient conditions and does not require any refrigeration. Finally, our results demonstrated the potential use of β–glucan and pomegranate juice bioactive films in diabetes and as an upcoming genuine candidate for dry powdered pharmaceuticals.

## Figures and Tables

**Table 1:** Coded and uncoded values of processing variables.

Independent Variables	Levels		
	−1	0	1
β–glucan (g), X_1_	0.5	1	1.5
Sodium alginate (g), X_2_	0.2	0.4	0.6
Pomegranate juice (mL), X_3_	10	20	30

**Table 2:** Experimental and coded values according to the Box-Behnken design.

Runs	β–Glucan		Sodium Alginate		Pomegranate Juice	
	Code	Experimental (g)	Code	Experimental (g)	Code	Experimental (mL)
1	−1	0.5	−1	0.2	0	20
2	−1	0.5	0	0.4	−1	10
3	−1	0.5	0	0.4	1	30
4	−1	0.5	1	0.6	0	20
5	0	1	−1	0.2	−1	10
6	0	1	−1	0.2	1	30
7	0	1	0	0.4	0	20
8	0	1	0	0.4	0	20
9	0	1	0	0.4	0	20
10	0	1	1	0.6	−1	10
11	0	1	1	0.6	1	30
12	1	1.5	−1	0.2	0	20
13	1	1.5	0	0.4	−1	10
14	1	1.5	0	0.4	1	30
15	1	1.5	1	0.6	0	20

**Table 3:** Preparation and composition of β–glucan, sodium alginate, pomegranate juice and glycerin in each film.

Run	β–Glucan Wet Weight (17.32% *w*/*w* Determined) (g)	Sodium Alginate (g)	Pomegranate Juice (12.42% *w*/*v* Determined) (mL)	Glycerin (25% *w*/*w* of the Total Solid Weight) (g)	Distilled Water (Up to Total Volume) (mL)	Sample Code
1	2.89 (0.5)	0.2	20	0.80	150	0.5-0.2-20
2	2.89 (0.5)	0.4	10	0.54	150	0.5-0.4-10
3	2.89 (0.5)	0.4	30	1.16	150	0.5-0.4-30
4	2.89 (0.5)	0.6	20	0.90	150	0.5-0.6-20
5	5.77 (1)	0.2	10	0.61	150	1-0.2-10
6	5.77 (1)	0.2	30	1.23	150	1-0.2-30
7	5.77 (1)	0.4	20	0.97	150	1-0.4-20-1
8	5.77 (1)	0.4	20	0.97	150	1-0.4-20-2
9	5.77 (1)	0.4	20	0.97	150	1-0.4-20-3
10	5.77 (1)	0.6	10	0.71	150	1-0.6-10
11	5.77 (1)	0.6	30	1.33	150	1-0.6-30
12	8.66 (1.5)	0.2	20	1.05	150	1.5-0.2-20
13	8.66 (1.5)	0.4	10	0.79	150	1.5-0.4-10
14	8.66 (1.5)	0.4	30	1.41	150	1.5-0.4-30
15	8.66 (1.5)	0.6	20	1.15	150	1.5-0.6-20

**Table 4:** Second order design matrix used to evaluate the effects of process variables and values of experimental responsefor films.

Run	Variables			Responses									
	β–Glucan (g) X_1_	Sodium Alginate (g) X_2_	Pomegranate Juice (mL) X_3_	Thickness (µm)	Density $\left(g\times {\mathrm{cm}}^{-3}\right)$	WVTR $\left(g\times h^{-1}\times m^{-2}\right)$	WVP $\left(g\times \mathrm{mm}\times {\mathrm{kPa}}^{-1}\times h^{-1}\times m^{-2}\right)$	Moisture Content (%)	Opacity $\left(\mathrm{Abs}\times {\mathrm{mm}}^{-1}\right)$	Solubility (Minutes)	L	a*	b*
1	0.5	0.2	20	88.6	1.53	28.3377	1.0565	17.59	4.75	3.42	29.51	0.23	1.7
2	0.5	0.4	10	64.4	1.46	25.0188	0.6780	17.72	7.86	2.5	29.18	0.02	1.26
3	0.5	0.4	30	132.4	1.69	32.1869	1.7932	17.86	2.77	4.2	32.11	0.35	1.54
4	0.5	0.6	20	129.7	1.55	31.0122	1.6925	15.23	1.46	2.34	27.83	−0.01	1.59
5	1	0.2	10	73.9	1.41	22.3472	0.6949	16.25	10.51	4.51	31.21	−0.02	1.16
6	1	0.2	30	138.7	1.66	28.2248	1.6473	16.20	1.81	5.38	31.86	0.47	1.63
7	1	0.4	20	135.2	1.57	27.8893	1.5866	13.98	3.55	4.07	33	0.26	1.76
8	1	0.4	20	133.7	1.52	27.7534	1.5614	14.02	3.84	4.41	33.07	0.28	1.71
9	1	0.4	20	130.5	1.59	28.8807	1.5859	13.89	3.82	5.19	33.13	0.34	1.81
10	1	0.6	10	112.7	1.45	24.6596	1.1694	12.93	5.47	3.11	33.36	0	1.5
11	1	0.6	30	147.3	1.71	30.1003	1.8657	15.04	3.03	4.25	32.59	0.44	1.76
12	1.5	0.2	20	134.8	1.60	26.2584	1.4894	13.92	4.07	5.22	33.31	0.2	1.56
13	1.5	0.4	10	116.6	1.42	23.8945	1.1723	12.31	5.08	6.33	33.43	−0.01	1.45
14	1.5	0.4	30	141.3	1.67	30.2471	1.7984	18.49	3.64	4.5	27.62	0.34	2.38
15	1.5	0.6	20	127.3	1.58	27.9846	1.4990	14.99	1.91	4.22	30.84	0.6	1.76

WVTR—Water Vapor Transmission Rate, WVP—Water Vapor Permeability, L—Lightness, a*—redness, b*—yellowness.

**Table 5:** Regression coefficients estimated of the quadratic model and their significance.

Independent Variables	Dependent Variables										
Term Regression Coefficients	Thickness (µm)		Density $\left(g\times {\mathrm{cm}}^{-3}\right)$	WVTR $\left(g\times h^{-1}\times m^{-2}\right)$	WVP $\left(g\times \mathrm{mm}\times {\mathrm{kPa}}^{-1}\times h^{-1}\times m^{-2}\right)$	Moisture Content (%)	Opacity $\left(\mathrm{Abs}\times {\mathrm{mm}}^{-1}\right)$	Solubility (Minutes)	L	a*	b*
Constant	b_0_	133.13	1.56	28.17	1.58	13.96	3.74	4.56	33.07	0.29	1.76
β–glucan (X_1_)	b_1_	13.11 *	ns	−1.02 *	0.09 *	−1.09 *	−0.26	0.97 *	0.82	0.06	0.13
Sodium alginate (SA) (X_2_)	b_2_	10.12 *	0.01	1.07 *	0.16 *	−0.72	−1.16 *	−0.57 *	−0.15	0.01	0.07
Pomegranate juice (X_3_)	b_3_	24.01 *	0.12 *	3.10 *	0.42 *	1.05 *	−2.21 *	0.23	−0.37	0.20 *	0.24*
β–glucan × SA (X_1_X_2_)	b_4_	−12.15 *	−0.01	−0.23	−0.15 *	0.85	0.28	0.017	−0.19	0.16 *	0.07
β–glucan × PJ (X_1_X_3_)	b_5_	−10.82 *	ns	−0.20	−0.12 *	1.51 *	0.91 *	−0.88 *	−2.19 *	ns	0.16
SA × PJ (X_2_X_3_)	b_6_	−7.55 *	ns	−0.10	−0.06 *	0.54	1.57 *	0.06	-0.35	−0.01	−0.05
β–glucan × β–glucan (X_1_^2^)	b_7_	−8.75 *	ns	0.86 *	−0.06 *	1.48 *	−0.52	−0.34	−2.18 *	−0.04	0.01
SA × SA (X_2_^2^)	b_8_	−4.28	ns	−0.64	−0.07 *	−0.01	−0.16	−0.41	−0.51	ns	−0.12
PJ × PJ (X_3_^2^)	b_9_	−10.70 *	ns	−1.20 *	−0.15 *	1.15 *	1.63 *	0.17	−0.29	−0.07	−0.12
*F*-value	62.28		9.98	30.92	109.58	7.29	12.34	6.51	1.85	4.35	2.83
*p*-value	0.0001		0.0104	0.0007	0.0001	0.0207	0.0064	0.0264	0.2573^ns^	0.0599^ns^	0.1323^ns^
R^2^	0.9912		0.9473	0.9824	0.9950	0.9292	0.9569	0.9214	0.7694	0.8868	0.8359

* *p* < 0.05, ns—not significant; b_0_—constant of the equation; b_1_, b_2_, b_3_—coefficients of the linear terms; b_4_, b_5_, b_6_—coefficients of the interaction effects; b_7_, b_8_, b_9_—coefficients of the quadratic effects; WVTR—Water Vapor Transmission Rate; WVP—Water Vapor Permeability; L—Lightness; a*—redness; b*—yellowness.

**Table 6:** The water activity of films made of β–glucan, sodium alginate, pomegranate juice and glycerin.

Sample Code	a_w_	Temperature of Measurement (°C)
0.5-0.2-20	0.2148 (0.009) ^b^	25.00
0.5-0.4-10	0.2266 (0.012) ^b^	25.03
0.5-0.4-30	0.2288 (0.005) ^ab^	25.07
0.5-0.6-20	0.2294 (0.007) ^ab^	25.04
1-0.2-10	0.2386 (0.006) ^ab^	25.02
1-0.2-30	0.2615 (0.009) ^ab^	24.99
1-0.4-20-1	0.2745 (0.013) ^a^	25.40
1-0.4-20-2	0.2344 (0.009) ^ab^	25.10
1-0.4-20-3	0.2274 (0.023) ^ab^	25.02
1-0.6-10	0.2508 (0.010) ^ab^	25.01
1-0.6-30	0.2447 (0.006) ^ab^	25.00
1.5-0.2-20	0.2418 (0.001) ^ab^	25.07
1.5-0.4-10	0.2566 (0.004) ^ab^	25.07
1.5-0.4-30	0.2285 (0.024) ^b^	25.00
1.5-0.6-20	0.2557 (0.004) ^ab^	25.03

Mean values of three replications and standard deviation in brackets. ^a,b^ different letters indicate significant differences between samples (*p* < 0.001).

**Table 7:** Predicted vs. actual values of the optimal film sample.

Film Composition	Predicted Values	Actual Values	Std. Dev.	SE Pred.
	0.98 g β–Glucan, 0.6 g SA, 14.80 mL PJ	1 g β–Glucan, 0.6 g SA, 14 mL PJ, 0.83 g Glycerin (1-0.6-14)		
Thickness (µm)	125.24	121.4	3.95	4.75
Density (g×cm^−m^)	1.49	1.47	0.03	0.04
WVTR (g×h^−×^×m^−m^)	26.3781	27.1224	0.61	0.74
WVP (g×mm×kPa^−×^×h^−h^×m^−m^)	1.394	1.390	0.04	0.05
Moisture content (%)	12.69	14.59	0.85	1.03
Opacity	3.38	4.48	0.82	0.99
Solubility (minutes)	3.44	4.53	0.50	0.61
L	32.72	36.45	1.63	1.97
a*	0.17	−0.05	0.11	0.13
b*	1.54	2.54	0.18	0.22
a_w_	nd	0.2287	nd	nd

Nd—undetermined; Std. Dev.—Standard deviation; SE Pred.—Standard error for predicting individual response.

## Data Availability

Not applicable.
